# On the Analogy between Natural Small-Pox, Inoculation, and Vaccination

**Published:** 1815-10

**Authors:** Richard Walker

**Affiliations:** of Oxford.


*For the London Medical and Physical Journal.*

### *On the Analogy between Natural Small-pox, Inoculation, and Vaccination;* by RICHARD WALKER, Esq. of Oxford.

A CIRCUMSTANCE which has lately occurred here has strongly excited my attention, and at the same time afforded me an opportunity of communicating what I consider to be new and not uninteresting, respecting the analogy between natural small-pox, inoculation, and vaccination.

It is proper to state, I was prepared and excited to this inquiry in consequence of reading a very excellent paper, by Mr. Hugo, of Crediton, on the subject of small-pox succeeding in two instances inoculation, and in several instances vaccination, where there was not the least reason to suspect it had been, in either instance, imperfectly conducted.

I shall briefly state here, that, in consequence of inoculation, (one instance only, I believe,) the natural small-pox was introduced into one part of Oxford, very numerous in population, during the hot weather of May last, many, of course, took it who had used no previous precaution; and, of those who had passed through vaccination perfectly, some few did not escape being seized with it, but which was of a comparatively mild kind; and one child, likewise, who had been inoculated, and had what was deemed by the practitioner who inoculated it a variolous eruption.* These facts correspond with those mentioned by Mr. Hugo as having happened at Crediton.

Among the instances of small-pox occurring after vaccination (a circumstance admitted as sometimes occurring, I presume, by every experienced practitioner,) were some in a family immediately under my own care, and which is the only instance of the kind which has happened to any patient of mine, to my knowledge; and which, in concurrence with other similar and corroborating circumstances, will constitute the chief subject of this paper.

Presuming that what has happened at Crediton and at Oxford, should a similar exciting cause occur, may and will

* I saw this child myself repeatedly during the latter affection, which was unquestionably genuine small-pox, and by no means of the most benign kind. This circumstance being considered as a phenomenon in practice, the child was seen by most of the professional gentlemen. With respect to the former affection, I was assured by the inoculator (an experienced practitioner) that the affection was unquestionably small-pox of a mild kind.

happen happen at other places, I shall proceed to the more immediate object of this paper.

I have lately had an opportunity of observing, in several instances, the rise, progress, and termination of the variolous eruption, the vesicle or pustule from inoculated small-pox, and of the cow-pox vesicle, or pustule, from vaccination, and have noticed so correspondent or striking a similarity between the whole, as to declare that no person acquainted with the variolous eruption can be at a loss to determine the identity of the genuine cow-pox pustule or vesicle. The essential character is the same in all three, and, allowing for an occasional difference, not constituting an essential character, they may be all considered in appearance the same. By giving, therefore, a short definition of the variolous pustule, the others must necessarily be identified.

The first appearance of a variolous eruption is a small inflamed spot, which, by the next day, will be found slightly elevated, by passing the finger over it. By the third or fourth day, and sometimes on the second day, this inflamed spot will have increased, principally in breadth or circumference, exhibiting upon its apex or center a minute white vesicle. The base continues increasing in inflammation, and extending in dimensions, till, on the seventh day or later, it has acquired its full size and maturity, having gradually changed from a minute vesicle, containing a thick, colourless, serous fluid, to an opake pustule, containing thickish matter, and by degrees turning brown and dry.*

The progress of the pustule from vaccination is briefly thus, and is so similar to the one from inoculation throughout, that, in practice, the description of one might serve for both, excepting that the former is ordinarily of a milder nature, and somewhat quicker in its progress.

1st. In about three or four days, the puncture should exhibit a slight degree of inflammation, and an elevation, perceptible under the finger, at the part; and by the fifth or sixth day, these having increased, with the addition of a minute vesicle at the apex.

2d. By the seventh or eighth day the vesicle should have increased so as to afford, on puncturing, a colourless serum,

* In the comparison between the eruptive variolous pustule, and the pustule immediately produced by inoculation and vaccination, the eruptive vesicle or pustule, on its first appearance, may be considered as equal with the first appearance of infection, having taken in the part inoculated or vaccinated, which is commonly about the third day, and here the comparison may commence.

or thin transparent fluid; and which is its fittest state for communicating the affection to another.

3d. By the tenth or eleventh day the vesicle will have increased in size, the efflorescent inflammation around the vesicle be at its height, and the vesicle itself beginning to turn brownish in the centre.

The indisposition which indicates the absorption of the virus into the constitution, occurs ordinarily about the time, or somewhat before, the efflorescence or inflammation of the arm is at its height, and lasts a day or two.*

4th. By the fifteenth day, viz. that day fortnight, inclusively, that is, including the day of vaccination, (which mode of reckoning is to be understood here throughout,) the pustule will have become completely turned and dry, and the whole terminated.

5th. The vaccinating serum or lymph should be taken from a perfect or genuine vesicle, at the time of its serous state, viz. on the eighth day, or a day before, or a day after, that is, before such vesicle is beginning to turn brown, or its fluid become opake.

6th. Such fluid should be inserted fresh, that is, before it has been suffered to become dry on the lancet, so as to require being moistened by warm water.†

7th. The operation should be performed thus:—the puncture should be so managed, as merely to occasion a very small effusion of moisture, but not that blood may follow, so as to endanger the vaccinating fluid being poured or washed out again, and the fluid from the lancet wiped, as it were, off the lancet into the wound.

With respect to a spurious vaccine pustule, having described the progressive appearance of the genuine one, and the circumstances on which the success of the operation depends, merely enforcing the propriety of adhering to the rule

* This indisposition consists in a similar affection to that which occurs in inoculation, but in a much milder degree, viz. lassitude, head-ach, giddiness, with nausea, but seldom (as is usually the case in inoculation) with vomiting, and a slight degree of feverish heat. This indisposition is ordinarily apparent in adults, but is such as might be passed over unnoticed in infants, by the attendants, if their attention be not particularly directed to it.

† I would not have it understood here that the lymph, having become dry, and requiring to be thus moistened, may not be effective; but that diluting the fluid, if possible, should be avoided. Moreover, I am solicitous to take the lymph myself, depending, then, in perfect confidence, on the due state of the lymph, and other circumstances.

of using such vaccinating fluid only as is in a serous state, as before mentioned, will be sufficient, believing that a deviation from this rule has been the chief source of what has been called the spurious or ineffective pustule.

In the spurious pustule arising from fluid taken too late, or kept too long, so as to be vitiated in its quality, the only species of spurious pustule which concerns those who propagate the infection from one human subject to another, the part inflames too suddenly, viz. to a considerable degree, by the second, third, or fourth day; and then as hastily vanishes, without acquiring the vesicular appearance so strongly characteristic of the genuine vesicle or pustule.

I am of opinion, that, should the indisposition escape notice, the due progress of the vaccinating part (including the efflorescent inflammation, or the areola, as it is sometimes called, which is more or less bright, according to the habit of the person,) will be sufficiently satisfactory, believing that under such circumstances the due indisposition must necessarily have occurred, but, perhaps, so slight, as to have passed unobserved, particularly in infants, who are chiefly the subjects of vaccination.

The local or inefficient pustule I consider to be that in which the areola or inflorescent inflammation is wanting; the pustule itself (though it may contain a serous fluid, capable of producing in another person an efficient pustule) passing through its progress in a languid manner, and of shorter duration, and owing either to an inactive state of the habit for receiving the infection, or to the imperfect manner of applying the virus, or to both conjointly.

Although it must be admitted that some few cases will occasionally occur in which persons who have passed through vaccination in a perfect manner, have afterwards been attacked by small-pox, I am apt to think that many of the failures we hear of are attributable to the neglect of attention to the due progress of the pustule, as above stated.

I have ascertained, as it appears to me, that in the cow-pox, as well as in small-pox, whether natural or from inoculation, there are two species,* viz. one of the milder kind, exhibiting pretty exactly the character of a variolous pustule, in what is called distinct small-pox, viz. the base circular, and well defined, compact, or but little diffused, and somewhat elevated; the vesicle at first not quite of a pale

* I have used the word *species* in conformity with custom; but it will be perceived that I consider the matter of small-pox as essentially the same in every instance, the difference being resolvable into what constitutes variety only.

white, white, and which, in its progress, turns of a brownish yellow, and at length to a dusky scab. The other species resembling the pustule in the confluent small-pox, and exhibiting, in character and appearance, a pretty exact similarity to the pustule in the confluent small-pox, and which is evidently of a less benign character than the former species: in this the pustule is somewhat irregular at its base, more diffused or spreading, and nearly flat; and the vesicle from the first of a pallid white, having occasionally a tint of bluish purple in it, exactly similar to what occurs occasionally in a pustule of the confluent kind of small-pox, and which, like that, turns in the end to a blackish or dark-coloured scab.*

There can be no doubt that both the above are only different species, or rather varieties, of the same affection, and depending chiefly upon the difference in the habit, since the mild, as well as the more virulent sort, will be productive each of the other occasionally by propagation, as is well known to be the case in variolous inoculation; though it rarely happens in inoculation that the distinct kind of small-pox is productive of the confluent. It may be observed, moreover, that in the more virulent or confluent sort of pustule, whether in natural small-pox, inoculation, or vaccination, the serous vesicle appears earlier, and, from its commencement, exhibits a higher degree of virulence than in the milder kind, and, in each instance, to the last, continues less elevated.

Moreover, I have had an opportunity of observing, even in persons of a single family, the different gradations from the most confluent or virulent kind of small pox to the mildest; and likewise the decided effects of vaccination in ameliorating the small-pox which followed. The family consisted of five children, three of whom I vaccinated four or five years ago, and all passed through it in the most satisfactory manner: the other two children, born since, had been neither inoculated nor vaccinated: one of the vaccinated children having been attacked by small-pox, I was desired to inoculate the rest, but it happened that all, excepting one, had previously taken the infection.

Premising that there are two kinds of small-pox, the distinct and confluent, as they are called, but which I consider specifically the same, the difference consisting in a less or greater degree of virulence only, there being a regular gradation from one to the other, the only difference worthy of

* This bluish colour is not to be confounded with *petechiæ*, or *purple spots*, as they are called, these appearing in the interstices of the pustules, and indicate a putrescent tendency.

notice between the eruptive pustule in the distinct or mild kind of small-pox, whether taken naturally or proceeding from inoculation, and the pustule produced at the part inoculated, is, that the latter does not acquire the perfectly hemispherical figure, when matured, which the former does, remaining always more or less flattened at the top, and, moreover, does not furnish a fluid of so perfectly a purulent nature.* In the mild kind of pustule produced by vaccination, the difference from inoculation pustule is ordinarily so slight as not to admit of distinction, and may be entirely disregarded in practice.

With respect to the more virulent kind, to which there is, in each instance, a regular gradation, this is marked (as in the confluent small-pox, so called,) in the pustule proceeding from vaccination, as in inoculation, in its being flatter, more diffused, and particularly in being more extended, and more irregular in its circumference; and, whilst in the state of vesicle, of a more pallid white. Observing, however, that the milder kind of pustule is more common in vaccination than in inoculation, seldom reaching that extreme degree of virulence which the latter does; nor is it accompanied by the eruptions which frequently surround the inoculation pustule;—all indications of its being an affection of a milder nature.

In the coloured Plate which is intended to exhibit the progressive difference between the pustule in inoculation and in

* I examined, in the course of their progress, the fluid contained in the pustules of the eruptive small-pox, taken in the natural way, and likewise those which were the effect of inoculation; and also the fluid contained in the pustule at the inoculated part, and in vaccination in each instance, from the perfectly serous state, on to dryness.

In the two former instances, the serous fluid changed to a perfectly purulent consistence and appearance, before it became dry and hardened; but not so in either of the two latter instances, remaining thin till nearly dry, when the fluid assumed the state or appearance of an inspissated solution of a pure gum.

Hence, the apparent difference between inoculation and vaccination is, that, in the former, the virus, after having entered and infected the system, is thrown off or exhausted, in part, at the place where the matter is inserted, in the form of efflorescent inflammation, and likewise by the variolous eruption; but, in vaccination, by the efflorescent inflammation alone. It is a common observation in inoculation, that, when the efflorescent inflammation is more than ordinarily violent, and, moreover, accompanied by pustules, that the variolous eruption is ordinarily proportionably diminished.

 vaccination, vaccination, that which is designed to represent the latter might serve for the mild kind in each instance; and the latter (taking away the extreme irregularity in the circumference, and the accompanying pustules, peculiar to the most virulent kind of inoculation pustule) to the more virulent sort of pustule in each.

*(To be continued.)*

### COLLECTANEA MEDICA,

